# Microorganisms in the human placenta are associated with altered CpG methylation of immune and inflammation-related genes

**DOI:** 10.1371/journal.pone.0188664

**Published:** 2017-12-14

**Authors:** Martha Scott Tomlinson, Paige A. Bommarito, Elizabeth M. Martin, Lisa Smeester, Raina N. Fichorova, Andrew B. Onderdonk, Karl C. K. Kuban, T. Michael O’Shea, Rebecca C. Fry

**Affiliations:** 1 Department of Environmental Sciences and Engineering, Gillings School of Global Public Health, University of North Carolina, Chapel Hill, North Carolina, United States of America; 2 Laboratory of Genital Tract Biology, Department of Obstetrics and Gynecology, Harvard Medical School and Brigham and Women’s Hospital, Boston, Massachusetts, United States of America; 3 Department of Pathology, Harvard Medical School and Brigham and Women’s Hospital, Boston, Massachusetts, United States of America; 4 Division of Pediatric Neurology, Department of Pediatrics, Boston Medical Center, Boston, Massachusetts, United States of America; 5 Department of Pediatrics, School of Medicine, University of North Carolina, Chapel Hill, North Carolina, United States of America; University of Michigan, UNITED STATES

## Abstract

Microorganisms in the placenta have been linked to adverse pregnancy outcomes as well as neonatal illness. Inflammation in the placenta has been identified as a contributing factor in this association, but the underlying biological mechanisms are not yet fully understood. The placental epigenome may serve as an intermediate between placental microbes and inflammation, contributing to adverse outcomes in the offspring. In the present study, genome-wide DNA methylation (n = 486,428 CpG sites) of 84 placentas was analyzed in relation to 16 species of placental microorganisms using samples collected from the Extremely Low Gestation Age Newborns (ELGAN) cohort. A total of n = 1,789 CpG sites, corresponding to n = 1,079 genes, displayed differential methylation (q<0.1) in relation to microorganisms. The altered genes encode for proteins that are involved in immune/inflammatory responses, specifically the NF-κB signaling pathway. These data support bacteria-dependent epigenetic patterning in the placenta and provide potential insight into mechanisms that associate the presence of microorganisms in the placenta to pregnancy and neonatal outcomes. This study lays the foundation for investigations of the placental microbiome and its role in placental function.

## Introduction

The placenta is a critical regulator of the prenatal environment and is essential for a healthy pregnancy and fetal development. It transports nutrients from mother to fetus and produces hormones necessary to maintain pregnancy and support the fetus [1]. The placenta can also harbor bacterial communities that, depending on their composition, affect pregnancy outcomes and fetal health [2, 3]. Previously it was thought that all bacteria in the placenta originate from infections of the lower genital tract [3], however, a number of studies have found that bacteria in the placenta are derived from vaginotropic non-infectious microflora [4, 5]. In addition, bacteria derived from other tissues may contribute to the placental microbiome, such as the oral cavity [6]. For example, it has been proposed that oral bacteria can translocate to the placenta by hematogenous transmission [7].

Importantly, certain bacteria in the placenta have been associated with deleterious pregnancy outcomes, such as preterm birth [3]. Among these placental bacteria are *Ureplasma urealyticum*, *Mycoplasma hominis*, *Gardnerella vaginalis*, and *Peptostreptococcus* sp., which are all associated with bacterial vaginosis, a disruption in the vaginal microbiota. Other placental bacteria, Group B *Streptococcus* and *Escherichia coli*, are associated with chorioamnionitis and fetal infection [3]. Little is known about the molecular responses to microorganisms in the placenta that potentially alter pregnancy and fetal outcomes.

The placental epigenome, particularly DNA methylation, is a potential mechanism that could explain this association and has not been explored. Epigenetic changes, such as DNA methylation, have been associated with bacteria in other tissues including *Helicobacter pylori* in the gastric mucosa [8–10], uropathogenic *Escherichia coli* in uroepithelial cells [11] and *Campylobacter rectus* in murine placental tissue [12]. In human placental cell culture, the presence of bacteria has been associated with both pro- and anti- inflammatory responses mediated by cytokines [13]. With an intriguing potential for sustained inflammation, our team showed in the Extremely Low Gestation Age Newborns (ELGAN) cohort that vaginotropic placental bacteria were associated with distinct inflammatory protein profiles in newborn blood with *Lactobacillus* sp. being anti-inflammatory and bacterial vaginosis-associated bacteria being pro-inflammatory [14]. These varying inflammatory responses induced by placental bacteria might be driven by CpG methylation in the placenta which could impact both placental function and fetal well-being.

To our knowledge, this is among the first studies to assess placental CpG methylation in relation to placenta bacteria. Here we investigated whether placental microbes were associated with altered placental DNA (CpG) methylation patterns in the ELGAN cohort. The goal of these analyses is to provide insights into how microorganimsms in the placenta alter the placental methylome and could thereby influence pregnancy and neonatal health outcomes.

## Methods

### ELGANs study subject recruitment

The recruitment process for the ELGAN study has been described in detail [15]. Briefly, between 2002 and 2004, we invited women who gave birth before 28 weeks gestational age at one of the 14 hospitals in 5 states in the United States to participate in the study. A total of 1,249 mothers and 1,506 infants enrolled in the study of which 1,365 placentas were collected and analyzed for microorganisms. A subcohort of 84 mother/infant pairs with similar average gestational and maternal age as the overall cohort were investigated in the present study (Table 1).

**Table 1:** Demographics.

	Overall with placenta microbiology (n = 1365)	Subcohort (n = 84)
	N (%)	N (%)
Fetal Sex		
Male	732 (54%)	58 (69%)
Female	633 (46%)	26 (31%)
Gestational age (weeks)	25.9 (23.0–27.9)	25.9 (23.7–27.9)
Maternal Age (years)	28.6 (13.2–47.3)	28.6 (16.0–40.6)
Maternal Race		
White	785 (58%)	38 (45%)
Non-white	562 (41%)	46 (55%)
NS	18 (1%)	0 (0%)
Delivery Method		
C-section	877 (64%)	61 (73%)
Natural	488 (36%)	23 (27%)
SES (insurance)		
Public	485 (36%)	35 (42%)
Private	741 (54%)	46 (55%)
Public & Private	46 (3%)	2 (2%)
Self-Pay Only	11 (1%)	1 (1%)
None	20 (1%)	0 (0%)
NS	62 (5%)	0 (0%)
UTI		
No	1090 (80%)	70 (83%)
Yes	203 (15%)	11 (13%)
NS	72 (5%)	3 (4%)
Vaginal Infection		
No	1120 (82%)	65 (77%)
Yes	173 (13%)	16 (19%)
NS	72 (5%)	3 (4%)
Antibiotic Use		
No	883 (65%)	61 (73%)
Yes	403 (30%)	19 (23%)
NS	79 (6%)	4 (5%)

NS = Not Specified

### Ethics statement

The Institutional Review Boards at Baystate Medical Center in Springfield, MA, Beth Israel Deaconess Medical Center in Boston, MA, Brigham and Women’s Hospital in Boston, MA, Children’s Hospital in Boston, MA, Massachusetts General Hospital in Boston, MA, Tufts New England Medical Center in Boston, MA, UMass memorial Medical Center in Worcester, MA, Yale-New Haven Hospital, New Haven, CT, Forsyth Hospital, Baptist Medical Center in Winston-Salem, NC, University Health Systems of Eastern Carolina in Greenville, NC, North Carolina Children’s Hospital in Chapel Hill, NC, DeVos Children’s Hospital in Grand Rapids, MI, Sparrow Hospital in Lansing, MI, University of Chicago Hospital in Chicago, IL, and William Beaumont Hospital in Royal Oak, MI approved all procedures. Informed, written consent was provided within a few days of delivery, either before or after. The mother’s consent covered both her and the child’s participation in the study.

### Placenta sample collection

Women participating in the ELGANs study were asked to provide their placentas for analysis. The technique used to collect the placentas is as follows: delivered placentas were placed in a sterile exam basin and transported to a sampling room where they were biopsied at the midpoint of the longest distance between the cord insertion and the edge of the placental disk. Using sterile technique, the amnion was pulled back to expose the chorion. Traction was applied to the chorion and the underlying trophoblast tissue and a piece of tissue was removed. The tissue was placed into a cryo-vial and immediately immersed into liquid nitrogen. Specimens were stored until processing at minus 80°C [4].

### Bacterial analysis of placenta

The placentas were biopsied as soon as possible after delivery and were assessed for microorganisms as described [4]. Briefly, a section of each placental specimen was removed using a sterile scalpel and homogenized in a phosphate buffered saline solution (PBS). Serial dilutions of the homogenate were made in PBS and aliquots of the original homogenate and the dilutions were plated onto selective and nonselective bacteriologic media, which included: prereduced Brucella base agar, tryptic soy agar, chocolate agar, and A-7 agar. Following incubation the various colony types were enumerated, isolated, and identified at the Brigham and Women’s Microbiology Laboratory using estimated criteria [16]. Since we have determined the constituents of the chorion parenchyma in the ELGANs prevent the reliable detection of bacterial DNA by PCR techniques, this study assessed only placental colonization patterns obtained by culture techniques.

### DNA extraction and assessment of DNA methylation

DNA extraction and assessment of DNA methylation by taking a 0.2 g subsection of placental tissue was cut from the frozen biopsy of dry ice, washed with sterile 1X PBS to remove residual blood, and homogenized with B-mercaptoethanol in Buffer RLT (Qiagen, Valencia, CA). DNA and RNA sequences with 18 or more nucleotides in length were collected using the AllPrep DNA/RNA/miRNA Universal Kit (Qiagen, Valencia, CA) following the manufacturer’s instructions. CpG methylation was assessed using the Illumina Human Methylation450 BeadChip^©^ array (Illumina, Inc., San Diego, CA). This asses the DNA methylation levels of 486,428 individual probes at single nucleotide resolution. Isolated DNA was bisulfate-converted using the EZ DNA methylation kit (Zymo Research, Irvine, CA) and converted DNA was hybridized onto the array. The DNA methylation data was collected at Expression Analysis, Inc. (Durham, NC; www.expressionanalysis.com).

Methylation levels were calculated and expressed as β values (β = intensity of the methylated allele (M) / (intensity of the unmethylated allele (U) + intensity of the methylated allele (M) + 100) [17]. Batch effect was not a significant source of variation, as determined by principle component analysis (PCA). Based on manufacturer recommendations, probes that had high detection P-values (P>0.1) were removed from analysis (n = 24,591). Following data filtration, data were normalized using the beta-mixture quantile (BMIQ) normalization method using the wateRmelon package (version 1.11.0) in R (version 3.2.3). After normalization, probes that were annotated as single nucleotide polymorphisms (SNPs) by Illumina were removed from further analysis (n = 84,121), leaving a total of 377,716 probes, representing 20,418 genes. The rationale for this exclusion is that SNP variation can lead to false readings of DNA methylation signals that may be attributed to genomic variation rather alterations in actual methylation levels [18].

### Statistical analysis

In order to determine whether the placental microbiome is associated with differences in DNA methylation patterning of the placenta, Analysis of Covariance (ANCOVA) was performed. There were 16 bacterial species assessed including: *Lactobacillus* sp., *Prevotella bivia*, *Gardnerella vaginalis*, anaerobic *Streptococcus*, *Peptostreptococcus* sp., *Escherichia coli*, alpha-hemolytic *Streptococcus*, *Ureaplasma urealyticum*, *Mycoplasma* sp., *Staphyloccocus* sp., *Propionibacterium* sp., *Actinomyces* sp., *Corynebacterium* sp., *Staphylococcus aureus*, *Streptococcus* Group B, and *Streptococcus* Group D. Each model examined whether presence or absence of an individual bacterial species or bacterial type was associated with differential placental methylation at any of the 377,716 probes. In the present study, variables were classified as confounding if they displayed an association with both the exposure, placental microorganisms, and the outcome, DNA methylation. Models were adjusted based on several dichotomous variables including fetal-sex, maternal race, whether the mother took an antibiotic during pregnancy, whether the mother experienced a vaginal infection during pregnancy, whether the mother experienced a urinary tract infection (UTI) during pregnancy, and whether the birth occurred via C-section. As there is no evidence that maternal age is associated with the presence of microorganisms in the placenta, it was not included as a confounding variable. Bacterial species were considered to be significantly associated with methylation if *p*-value <0.05 and the false discovery rate-corrected *p*-value <0.1 for any given probe. Data analysis was carried out using Partek Genomic Suites 6.6.

### Gene set-based analysis

The differentially methylated probes and corresponding genes were analyzed to determine if they were enriched for specific biological functions. Four gene sets were analyzed where gene content was established using www.uniprot.org. The four gene sets that were tested were selected for their known roles in fetal development: transport-related genes (n = 3,704) [19], immune-related genes (n = 1,622) [19–21], inflammation-related genes (n = 410) [20,22] and growth/transcription factor-related genes (n = 2030) [23–25]. A χ^2^ test was used to identify enrichment of key processes. Once the enriched processes were identified a right-tailed Fisher Exact test (α = 0.05) was conducted on the significant genes in the enriched pathways to identify enriched canonical pathways and transcription factors using Ingenuity Pathway Analysis software as described in Martin *et al*., 2015 [26].

## Results

### Study subject characteristics

The placentas of 84 subjects within the ELGAN cohort were analyzed for this study. The average gestational age and maternal age of the subcohort of 84 are the same as the overall cohort (n = 1,365) (Table 1). There are some differences between the subcohort and the overall cohort, including maternal race and sex of the infant. The overall cohort is predominately white (58%) while the subcohort is only 45% white and 55% non-white. The majority of the infants in both the overall cohort and subcohort are males. However, the percentage of males in the subcohort (69%) is much larger than the percentage in the overall cohort (45%). Of the 84 infants, 23 (27%) were delivered vaginally, while 61 (73%) were delivered by Cesarean section. Of these, 37 (44%) were on public insurance and 46 (55%) were on private insurance. A total of 11 (13%) of the women had a urinary tract infection, 16 (19%) had a vaginal infection, and 19 (22%) used an antibiotic during pregnancy.

The most common bacteria present in the study placentas was *Propionbacterium* sp. which was detected in 10 (11.9%) placentas (Table 2). This was followed closely by *Staphylococcus aureus* which was detected in seven (8.3%) placentas. *Ureaplasma urealyticum* was detected in five (6.0%) and *Actinomyces* was detected in four (4.8%). *Lactobacillus* sp. was detected in three (3.6%) of placentas and *Escherichia coli* was detected in two (2.4%) placentas. The majority of the microorganisms were present in two to three placentas and 44 (52%) of the placentas had no detectable microorganism.

**Table 2:** Presence of microorganisms in the placenta and methylation of CpG probes and associated genes.

		CpG Probes			Genes		
Bacteria	n (%)	#	# HyperM^1^ N (%)	# HypoM^2^ N (%)	#	# HyperM^1^ N (%)	# HypoM^2^ N (%)
*Lactobacillus* sp.	3 (3.6)	44	10 (23)	34 (77)	32	6 (19)	26 (81)
*P*. *bivia*	3 (3.6)	63	13 (21)	50 (79)	49	12 (24)	37 (76)
*G*. *vaginalis*	3 (3.6)	53	16 (30)	37 (70)	43	11 (26)	32 (74)
Anaerobic *Streptococcus*	4 (4.8)	0	--	--	0	--	--
*Peptostreptococcus* sp.	3 (3.6)	13	1 (8)	12 (92)	11	1 (9)	10 (91)
*E*. *coli*	2 (2.4)	53	24 (45)	29(55)	24	6 (25)	18 (75)
Alpha *Streptococcus*	3 (3.6)	167	35 (21)	132 (79)	105	13 (12)	92 (88)
*U*. *urealyticum*	5 (6.0)	21	11 (52)	10 (48)	11	6 (55)	5 (45)
*Mycoplasma* sp.	9 (10.7)	0	--	--	0	--	--
*Staphylococcus* sp.	7 (8.3)	40	25 (63)	15 (37)	22	8 (36)	14 (64)
*Propionibacterium* sp.	10 (11.9)	2	2 (100)	0 (0)	1	1 (100)	0 (0)
*Actinomyces* sp.	4 (4.8)	2	0 (0)	2 (100)	1	0 (0)	1 (100)
*Corynebacterium* sp.	3 (3.6)	24	2 (8)	22 (92)	18	1 (6)	17 (94)
*Staphylococcus aureus*	7 (8.3)	40	25 (63)	15 (37)	22	8 (36)	14 (64)
*Streptococcus* Group B	2 (2.4)	1,257	28 (2)	1,229 (98)	802	12 (1)	790 (99)
*Streptococcus* Group D	2 (2.4)	206	128 (62)	78 (38)	112	54 (48)	58 (52)

^1^ HyperM = Hypermethylated

^2^ HypoM = Hypomethylated

### Identification of differentially methylated probes in ELGAN placentas exposed to bacteria

**Fig 1:**
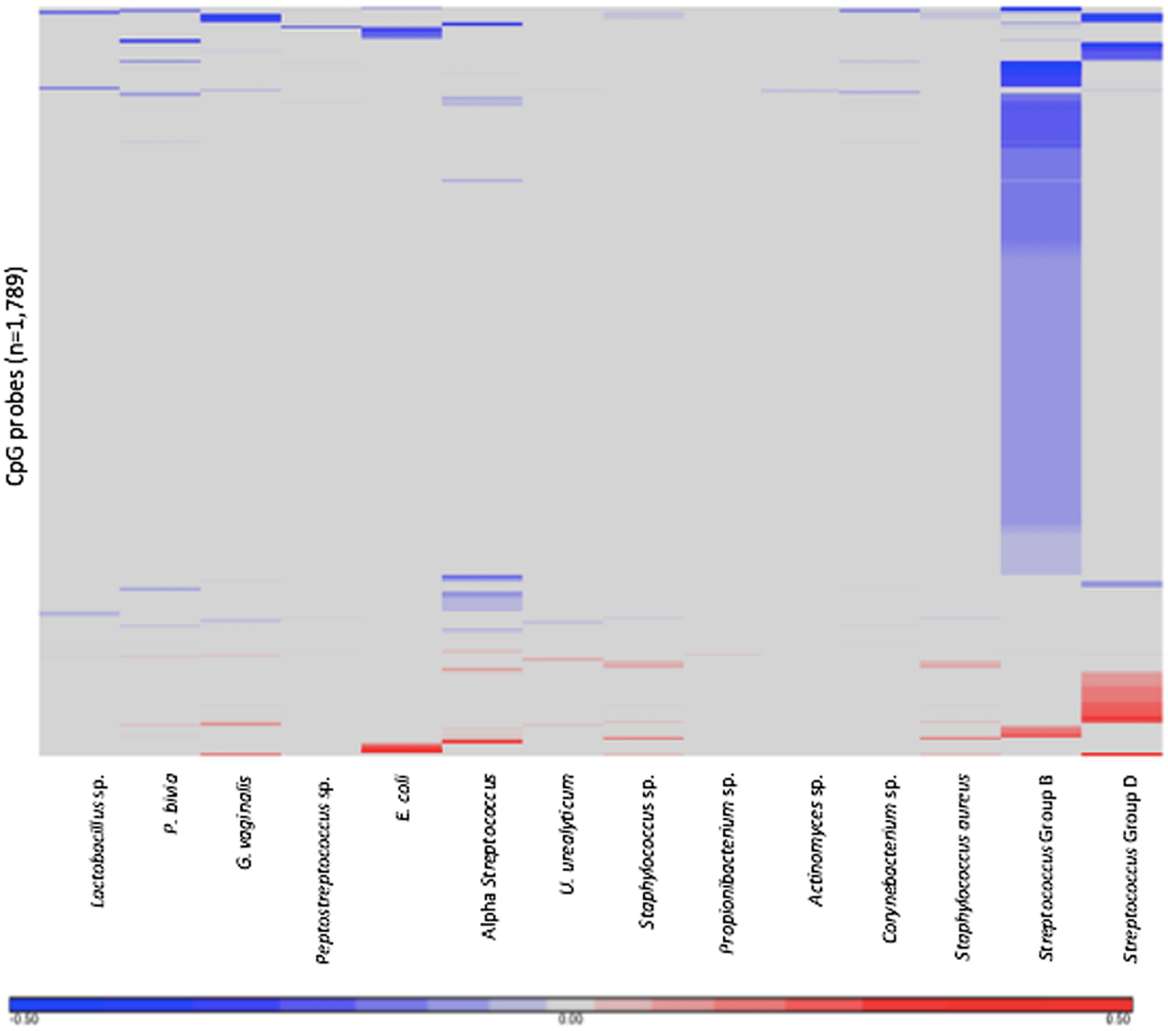
Heatmap of differentially methylated CpG probes corresponding to each of the microorganisms. The heatmap displays the 1,789 differentially methylated probes in relation to 17 microorganisms. Red represents increased methylation and blue represents decreased methylation.

Placental CpG methylation differences were analyzed within the ELGAN cohort between 84 individuals with or without placental microbes for 377,716 CpG probes representing 20,418 genes. Statistical significance was adjusted using false discovery rate-corrected p-values (q<0.1). Of the 16 microbial species all but two, namely anaerobic *Streptococcus* and *Mycoplasma* sp., were associated with differentially methylated probes. A total of 1,789 probes, corresponding to 1,079 genes, were significantly differentially methylated between placentas that harbored bacteria and placentas that did not (S1 File). The probes that displayed differential methylation were unique depending on which bacteria was present (Fig 1).

The microbial species that was associated with the greatest number of altered CpG sites was *Streptococcus* Group B with 1,257 differentially methylated CpG probes (n = 802 genes) (Table 2). The methylation pattern for *Streptococcus* Group B leans heavily toward hypomethylation with 98% (1,232) of the sites being hypomethylated.

Following *Streptococcus* Group B, the two microbial species that were associated with the most differentially methylated probes were *Streptococcus* Group D and alpha-hemolytic *Streptococcus* with 206 and 167 CpG probes, respectively. Most of the probes altered in relation to *Streptococcus* Group D were hypermethylated (62%) while alpha-hemolytic *Streptococcus*-associated probes were mostly hypomethylated (79%). The CpG probes correspond to 112 genes for *Streptococcus* Group D and 105 genes for alpha-hemolytic *Stretptococcus*. The presence of *E*.*coli* was associated with 53 CpG probes representing 43 different genes. Of those probes 55% were hypomethylated.

### Location of differentially methylated probes

The majority of the significant CpG probes for all 14 bacterial species were located in the body of the gene (Table 3). Both of the probes associated with *Propionibacterium* sp. were located in the gene body. *Peptostreptococcus* sp. had the next highest percentage of differentially methylated probes in the gene body at 62%, while *Actinomyces* sp. had the lowest percentage at 22%. There were also a large percentage of probes that did not correspond with a gene and therefore do not have a gene location. The remaining probes fell into the 1^st^ exon, 3’UTR, 5’UTR, TSS1500, and TSS200 locations.

**Table 3:** Gene-Specific location of CpG methylation.

Bacteria	1^st^ exon N (%)	3’UTR N (%)	5’UTR N (%)	Body N (%)	TSS1500 N (%)	TSS200 N (%)	N/A N (%)	# probes
*Lactobacillus* sp.	2 (5)	2 (5)	0 (0)	26 (59)	3 (6)	2 (5)	9 (20)	44
*P*. *bivia*	0 (0)	4 (6)	3 (5)	28 (44)	7 (11)	8 (13)	13 (21)	63
*G*. *vaginalis*	0 (0)	5 (9)	5 (9)	31 (59)	2 (4)	3 (6)	7 (13)	53
*Peptostreptococcus* sp.	0 (0)	0 (0)	0 (0)	8 (62)	2 (15)	1 (8)	2 (15)	13
*E*. *coli*	1 (2)	2 (4)	3 (6)	12 (22)	10 (19)	4 (7)	21 (40)	53
Alpha *Streptococcus*	8 (5)	8 (5)	10 (6)	72 (43)	17 (10)	11 (6)	41 (25)	167
*U*. *urealyticum*	0 (0)	0 (0)	5 (24)	6 (28)	0 (0)	5 (24)	5 (24)	21
*Staphylococcus* sp.	1 (3)	3 (7)	2 (5)	14 (35)	6 (15)	3 (7)	11 (28)	40
*Propionibacterium* sp.	0 (0)	0 (0)	0 (0)	2 (100)	0 (0)	0 (0)	0 (0)	2
*Actinomyces* sp.	0 (0)	0 (0)	0 (0)	1 (50)	0 (0)	0 (0)	1 (50)	2
*Corynebacterium* sp.	0 (0)	1 (4)	0 (0)	13 (54)	3 (13)	1 (4)	6 (25)	24
*Staphylococcus aureus*	1 (3)	3 (7)	2 (5)	14 (35)	6 (15)	3 (7)	11 (28)	40
*Streptococcus* Group B	33 (3)	50 (4)	107 (9)	547 (43)	148 (12)	66 (5)	306 (24)	1,257
*Streptococcus* Group D	9 (4)	14 (7)	22 (11)	56 (27)	33 (16)	34 (17)	38 (18)	206

### Enrichment of biological functions and pathways among the differentially methylated gene sets

We further analyzed whether these differentially methylated probes in the placenta were enriched for four specific biological functions: inflammation, growth/transcription factors, transport, and/or immune response, selected for their known critical functions for fetal development. For this analysis, the unique genes that corresponded with differentially methylated probes were considered (n = 1,080). A Yates corrected χ^2^ test was used in conjunction with a right-tailed Fisher Exact test (α = 0.05) to determine that immune-related proteins (n = 50, p = 0.000075), transcription/growth factors (n = 134, p = 0.00173), and inflammatory response (n = 35, p = 1.27E-09) were enriched (S1 Table).

Among the 50 immune-related genes, Nuclear Factor Kappa-light-chain-enhancer of activated B Cells (NF-κB) signaling was the top canonical pathway (p = 5.98E-05) (S1 Table). Five of the immune-related genes are involved in NF-κB signaling including two kinases, Bone Morphogenetic Protein Receptor Type 1A (*BMPR1A*) and Protein Kinase C Zeta (*PRKCZ*), two enzymes, TNF Alpha Induced Protein 3 (*TNFAIP3*) and Ubiquitin Conjugating Enzyme E2 N (*UBE2N*), and one transmembrane receptor, Insulin Like Growth Factor 1 Receptor (*IGF1R*). The CpG probes corresponding to these five genes were all hypomethylated. All five of the genes had at least one of their hypomethylated probes altered when *Streptococcus* Group B bacteria was present in the placenta. Alpha-hemolytic *Streptococcus* was associated with a hypomethylated probe for *BMPR1A* and *PRKCZ* while *IGF1R* had probes altered with the presence of *P*. *bivia*. Based on the transcription factor occupancy theory, which is a proposed mechanism of genome-wide patterning of DNA methylation [27], we identified the transcription factors of the immune-related genes that were enriched. The top five transcription factors were: Tripartite Motif Containing 24 (*TRIM24)* (p = 7.27E-05), RELA Proto-Oncogene NF-κB Subunit (*RELA*) (p = 2.54E-04), Tumor Protein P53 (*TP53*) (p = 3.18E-04), Core-binding Factor Beta Subunit (*CBFB*) (p = 6.58E-04), and POU Class 5 Homeobox 1 (*POU5F1*) (p = 7.82E-04) (S2 Table).

## Discussion

The presence of microorganisms in the placenta has been associated with inflammation and negative birth outcomes, including preterm birth [3]. Intriguing data from mouse studies and cell culture suggest that bacteria in the placenta can alter CpG methylation [12, 13]. To evaluate whether the presence of microorganisms is associated with the human placental epigenome, we integrated data for 16 different microbial species in the placenta with genome-wide CpG methylation levels. A total of 1,789 probes, representing 1,079 genes, were identified that were differentially methylated (q<0.1) in relation to placental bacteria. Interestingly, each bacteria type corresponded with a distinct CpG methylation pattern within the placenta. The genes that corresponded to the differentially methylated probes were enriched for their roles as growth/transcription factors, immune response proteins, and inflammatory response proteins and are active in the NF-κB pathway. Interestingly, the NF-κB pathway is critical during pregnancy and for fetal development [28, 29]. Overall, our findings demonstrate that the presence of microorganisms in the placenta is associated with differences in CpG methylation. The specific genes with altered methylation could represent etiologic factors that contribute to placental function and fetal health.

The data from the study show that 14 of the bacteria species were associated with unique CpG probes, with not much overlap between the bacterial types. The three *Streptococcus* sp. displayed the most differentially methylated CpG probes. The presence of *Streptococcus* Group B was associated with the most differentially methylated probes, corresponding to 802 genes. *Streptococcus* Group D and α-*Streptococcus* followed with 206 probes (112 genes) and 167 probes (105 genes), respectively. *Propionibacterium* sp. was the most prevalent bacteria present in 10 placentas. The majority of the differentially methylated probes for each placental bacterium, were hypomethylated, meaning they displayed decreased methylation levels at a specific probe in relation to microbes. The fact that there is little overlap between the CpG probes that are differentially methylated for each bacterium shows a diverse placental epigenetic response depending on which bacterial species is present.

In the present analysis, the NF-κB pathway was enriched among the microorganism-associated differentially methylated genes. The NF-κB pathway is a pro-inflammatory signaling pathway that is activated by pathogens or stressors [30]. It has been shown that bacterial plasmid DNA activates a signaling cascade that leads to activation of the NF-κB pathway and expression of inflammatory genes [31]. There is also accumulating evidence that NF-κB activity increases with the onset of labour [32, 33] and is tied to children’s health later in life [34]. Of the five NF-κB genes that were differentially methylated, four are known to induce NF-κB signaling. For example, *UBE2N* plays a role in activating the NF-κB pathway [35] and *PRKCZ* is a component of the TNF/IL1β pathway that controls activation of the NF-κB pathway [36–38] and induces contraction of myometrial tissue during late pregnancy [39]. *TNFAIP3* is the only differentially methylated gene that inhibits NF-κB activation [40, 41]. All probes associated with the five NF-κB genes were hypomethylated, with at least one of the probes being altered in the presence of *Streptococcus* Group B. While there are exceptions, hypomethylation of CpG sites often leads to the upregulation of genes [42]. From this evidence, we conclude that the presence of microorganisms in the placenta, especially *Streptococcus* Group B, is likely associated with activation of the NF-κB pathway. These findings are in agreement with the positive association between anaerobic *Streptococcus* and systemic inflammation in the ELGANs in early life, where these bacteria were associated with increased levels of five out of 16 tested inflammatory proteins that are upregulated by NF-κB activation including, IL-1β, IL-6, TNFR1, TNFR2, and E-selectin [14].

The transcription factor occupancy theory proposes that transcription factors are drivers of gene-specific DNA methylation patterns [27]. The transcription factor binding either prevents or allows the DNA methylation machinery access to the DNA sequence and therefore this binding influences gene-specific methylation. Thus, to test this theory, an analysis was carried out to identify the enriched transcription factors of the immune-related genes. One of the enriched transcription factors was NF-κB p65 (RelA), a transcriptional activator of the NF-κB pathway. Six of our 50 immune-related genes are transcribed by RelA and the CpG probes that are associated with these genes were all hypomethylated in the presence of *Streptococcus* Group B bacteria. RelA is involved in the expression of IL-8, which is a chemokine that mediates an inflammatory response. An increase in IL-8 has been associated with premature labor [43]. RelA has also been identified as a key regulator of the cytokine environment that is required for a successful pregnancy. The suppression of RelA is critical for the shift towards Th2-type immune responses during pregnancy [44, 45]. POU5F1 was associated with all hypomethylated genes and is of interest because it plays a crucial role in embryonic development and stem cell pluripotency [46]. These enriched transcription facts may influence CpG methylation in the placenta as well as pregnancy outcomes and fetal development.

There are multiple factors that should be considered when interpreting the results from this study. The sample size was relatively small (n = 84) and bacteria prevalence was low. Nevertheless, 52% of the placentas in the study harbored at least one type of bacteria, which is similar to a prior study of 1,083 placentas in the ELGAN cohort that found 79% and 43% of preterm placentas at 23 weeks and 27 weeks, respectively, carried microorganisms [4]. It is important to note that the data on microbial presence represent live, functional bacteria detected as colony forming units rather than simply DNA, which may be derived from dead or non-functional microorganisms. While differential methylation of CpG sites in placentas with microorganisms was identified, gene expression was not a part of the analysis. As a proxy for gene expression data the placental methylome data was integrated into existing genomics datasets to establish the functional epigenetics and biological pathways. Future research should have a larger sample size and incorporate mRNA and protein expression data along with the CpG methylation data.

This study is among the first to investigate the potential effect of placental bacteria on the methylome of the placenta. While outside the scope of the current study, future analysis could examine the molecular mechanism underlying microbe-CpG methylation, which could include altered DNA methyltransferase activity [11, 47, 48]. A major observation from the current study is that the CpG methylation patterning differs depending on microorganism presence in the placenta. These differences might be associated with variation in fetal development, birth outcomes and later life disease of premature babies. The genes corresponding to the differentially methylated probes in this study are associated with immune and inflammatory responses, especially the NF-κB pathway. These epigenetic changes at the maternal-fetal interface could have long-lasting consequences for the health of the individual.

## Supporting information

S1 File β-differences of statistically significant differentially methylated probes for each bacterial species. (XLS)

S1 Table Genes associated with enriched biological functions and associated canonical pathways. Genes that were differentially methylated in the present study and were involved in the enriched biological functions are listed along with the top five enriched canonical pathways for each gene list and associated p-values.
(DOCX)

S2 Table Enriched transcription factors and associated genes from gene set. (DOCX)
