# Supplementary material for: Microorganisms in the human placenta are associated with altered CpG methylation of immune and inflammation-related genes
Source: PLoS One. 2017 Dec 14;12(12):e0188664. doi: 10.1371/journal.pone.0188664 (PMC5730116; doi:10.1371/journal.pone.0188664)
Supplement: S1 Table — Genes that were differentially methylated in the present study and were involved in the enriched biological functions are listed along with the top five enriched canonical pathways for each gene list and associated p-values. (DOCX) [file pone.0188664.s002.docx]

**S1 Table. Genes associated with enriched biological functions and associated canonical pathways**

| **Immune-Related**  (n=50) | **Transcription/Growth Factors**  (n=134) | **Inflammatory response**  (n=35) |
| --- | --- | --- |
| \| *ABL1* \| \| --- \| \| *ADAR* \| \| *ARHGEF2* \| \| *ATG5* \| \| *BMPR1A* \| \| *C4BPA* \| \| *CACNA1C* \| \| *CCL28* \| \| *CD4* \| \| *CHGA* \| \| *CLEC4C* \| \| *COL4A3BP* \| \| *CSF1R* \| \| *DAB2IP* \| \| *DDX60* \| \| *DEFA6* \| \| *EIF2AK4* \| \| *GGT1* \| \| *HLA-H* \| \| *IGF1R* \| \| *IL4R* \| \| *IRF4* \| \| *IRF7* \| \| *LAX1* \| \| *LGALS3BP* \| \| *LY86* \| \| *NLRP1* \| \| *NPY* \| \| *ORAI1* \| \| *PRELID1* \| \| *PRKCZ* \| \| *PTK2B* \| \| *PVRL1* \| \| *PYCARD* \| \| *RNF135* \| \| *SFTA3* \| \| *SKAP2* \| \| *SLC39A4* \| \| *SMAD6* \| \| *SPACA3* \| \| *SRC* \| \| *TNFAIP3* \| \| *TNK2* \| \| *TOLLIP* \| \| *TRIM26* \| \| *TRIM27* \| \| *TYRO3* \| \| *UBE2N* \| \| *UNC5B* \| \| *ZNF683* \| | \| *EGFR* \| \| --- \| \| *FGFR2* \| \| *IGF1R* \| \| *FGF2* \| \| *ABTB1* \| \| *TNK2* \| \| *ABL1* \| \| *ADD1* \| \| *AKAP13* \| \| *PRMT2* \| \| *ADRA2C* \| \| *AKT2* \| \| *TFAP2A* \| \| *AP2M1* \| \| *ANKFY1* \| \| *ANKRD27* \| \| *ANKS1A* \| \| *API5* \| \| *ARID1B* \| \| *ARHGEF11* \| \| *ASCL1* \| \| *BAIAP2* \| \| *BMP7* \| \| *BCAR1* \| \| *BMP8B* \| \| *BMPR1A* \| \| *CDKN1C* \| \| *CELSR3* \| \| *CHSY1* \| \| *CREG1* \| \| *CSNK2B* \| \| *DAB2IP* \| \| *DOCK1* \| \| *DRG1* \| \| *DNM2* \| \| *DPF3* \| \| *EIF2AK4* \| \| *ENG* \| \| *EGR4* \| \| *EHD1* \| \| *EGFL8* \| \| *EGF* \| \| *EPS15* \| \| *ERCC1* \| \| *EPS8L1* \| \| *FBN3* \| \| *PTK2B* \| \| *FGF14* \| \| *FEZ1* \| \| *FOXK1* \| \| *GAS7* \| \| *GDF5* \| \| *GDF9* \| \| *GRHL2* \| \| *GSK3B* \| \| *GXYLT2* \| \| *HYAL1* \| \| *IGFBP7* \| \| *HTRA1* \| \| *IGFBP4* \| \| *INPP5K* \| \| *ITGB5* \| \| *IRS2* \| \| *CAMK1D* \| \| *KCNC1* \| \| *PRKCZ* \| \| *RPS6KA2* \| \| *KSR1* \| \| *LEFTY2* \| \| *TRIM71* \| \| *LRP1* \| \| *LEFTY1* \| \| *MCM7* \| \| *MAPK12* \| \| *MAP2K5* \| \| *SBF1* \| \| *NAIF1* \| \| *NCK2* \| \| *SLC9A3R1* \| \| *NTN1* \| \| *NPHP4* \| \| *SLC34A2* \| \| *NUBP1* \| \| *PA2G4* \| \| *PCSK6* \| \| *PAK4* \| \| *PARD3* \| \| *GIGYF1* \| \| *PLXNA1* \| \| *PPP1CA* \| \| *PSPN* \| \| *PTPRJ* \| \| *RAB35* \| \| *RERE* \| \| *RAB11FIP2* \| \| *RHBDF1* \| \| *RPTOR* \| \| *POLR2B* \| \| *RUVBL1* \| \| *SASH3* \| \| *SIK1* \| \| *SMAD6* \| \| *SMAD7* \| \| *SKI* \| \| *SFRP5* \| \| *SHC1* \| \| *SOX10* \| \| *SLC9A1* \| \| *SOX5* \| \| *SPSB4* \| \| *SSH1* \| \| *STAT5A* \| \| *SPTB* \| \| *SRC* \| \| *STEAP3* \| \| *TAOK3* \| \| *TEAD4* \| \| *TGFB3* \| \| *PLAT* \| \| *TSC2* \| \| *USP4* \| \| *TYRO3* \| \| *USP9X* \| \| *VASN* \| \| *SORBS3* \| \| *WNT7A* \| \| *WWOX* \| \| *WFS1* \| \| *WWP2* \| \| *HIVEP3* \| \| *ZFHX3* \| \| *ZIC1* \| \| *EEF1G* \| \| *CSF1R* \| | *ABL1*  *ADAR*  *ATG5*  *BMPR1A*  *CACNA1C*  *CCL28*  *CD4*  *CHGA*  *CSF1R*  *DDX60*  *DEFA6*  *EIF2AK4*  *GGT1*  *IGF1R*  *IL4R*  *IRF4*  *IRF7*  *LAX1*  *LGALS3BP*  *NECTIN1*  *NPY*  *ORAI1*  *PRKCZ*  *PTK2B*  *PYCARD*  *SLC39A4*  *SPACA3*  *SRC*  *TNFAIP3*  *TOLLIP*  *TRIM26*  *TRIM27*  *TYRO3*  *UBE2N*  *UNC5B* |
| **Associated Canonical Pathways** (P-values) | | |
| -NF-κB Signaling (5.98E-05)  -Role of Osteoblases, Osteoclasts and Chondrocytes in Rheumatoid Arthritis (1.97E-04)  -Molecular Mechanisms of Cancer (2.17E-04)  -Calcium-induced T Lymphocyte Apoptosis (4.84E-04)  -CCR5 Signaling in Macrophages (5.52E-04) | -Molecular Mechanisms of Cancer (2.82E-13)  -Human Embryonic Stem Cell Pluripotency (3.79E-13)  -Role of Osteoblasts, Osteoclasts and Chondrocytes in Rheumatoid Arthritis (2.23E-11)  -Axonal Guidance Signaling (7.02E-11)  -Glioma Signaling (1.10E-10) | -NF-κB Signaling (1.09E-05)  -Calcium-induced T Lymphocyte Apoptosis (1.72E-04)  -CCR5 Signaling in Macrophages (1.96E-04)  -STAT3 Pathway (2.41E-04)  -Macropinocytosis Signaling (3.15E-04) |
