# Supplementary material for: Microorganisms in the human placenta are associated with altered CpG methylation of immune and inflammation-related genes
Source: PLoS One. 2017 Dec 14;12(12):e0188664. doi: 10.1371/journal.pone.0188664 (PMC5730116; doi:10.1371/journal.pone.0188664)
Supplement: S2 Table — (DOCX) [file pone.0188664.s003.docx]

**S2 Table. Enriched transcription factors and associated genes from gene set**

| Transcription Factor | Associated Genes |
| --- | --- |
| Tripartite Motif Containing 24 (TRIM24) | Tripartite Motif Containing 26 (*TRIM26*)  Galectin 3 Binding Protein (*LGALS3BP*)  Interferon Regulatory Factor 7 (*IRF7*)  DExD/H-Box Helicase 60 (*DDX60*) |
| RELA Proto-Oncogene,  NF-κB Subunit (RELA) | Collagen Type IV Alpha 3 Binding Protein (*COL4A3BP*)  Insulin Like Growth Factor 1 Receptor (*IGF1R*)  Interferon Regulatory Factor 4 (*IRF4*)  Interferon Regulatory Factor 7 (*IRF7*)  ORAI Calcium Release-Activated Calcium Modulator 1 (*ORAI1*)  TNF Alpha Induced Protein 3 (*TNFAIP3*) |
| Tumor Protein p53 (TP53) | Rho/Rac Guanine Nucleotide Exchange Factor 2 (*ARHGEF2*)  Colony Stimulating Factor 1 Receptor (*CSF1R*)  Insulin Like Growth Factor 1 Receptor (*IGF1R*)  Interleukin 4 Receptor (*IL4R*)  Interferon Regulatory Factor 7 (*IRF7*)  PRELI Domain Containing 1 (*PRELID1*)  Protein Kinase C Zeta (*PRKCZ*)  PYD and CARD Domain Containing (*PYCARD*)  SMAD Family Member 6 (*SMAD6*)  SRC Proto-Oncogene, Non-Receptor Tyrosine Kinase (*SRC*)  Unc-5 Netrin Receptor B (*UNC5B*) |
| Core-Binding Factor Beta Subunit (CBFB) | CD4 Molecule (*CD4*)  Colony Stimulating Factor 1 Receptor (*CSF1R*)  Galectin 3 Binding Protein (*LGALS3BP*) |
| POU Class 5 Homeobox 1 (POU5F1) | Bone Morphogenetic Protein Receptor Type 1A (*BMPR1A*)  Insulin Like Growth Factor 1 Receptor (*IGF1R*)  ORAI Calcium Release-Activated Calcium Modulator 1 (*ORAI1*)  PYD and CARD Domain Containing (*PYCARD*)  SRC Proto-Oncogene, Non-Receptor Tyrosine Kinase (*SRC*) |
